# Very low prevalence of bovine tuberculosis in cattle in Sylhet district of Bangladesh

**DOI:** 10.1016/j.heliyon.2023.e22756

**Published:** 2023-11-20

**Authors:** Pradeep Kumar Mandal, Md. Irtija Ahsan, Hrithik Deb Apu, Sharmin Akter, Syed Sayeem Uddin Ahmed, Suman Paul

**Affiliations:** a Lahan Technical School, Lahan, 56502, Nepal; b Department of Epidemiology and Public Health, Sylhet Agricultural University, Sylhet, 3100, Bangladesh

**Keywords:** Bovine tuberculosis (bTB), *Mycobacterium bovis*, Caudal Fold Tuberculin (CFT) test, Prevalence, Distribution

## Abstract

Bovine tuberculosis (bTB), a chronic zoonotic disease in cattle, has a substantial socio-economic and public health impact. This study was conducted to estimate the prevalence and geographical distribution of bTB in the Sylhet district of Bangladesh. A cross-sectional study was conducted at all 12 upazilas of Sylhet district, which included 512 randomly selected cattle from 48 farms. Selected animals were tested with the Caudal Fold Tuberculin (CFT) test to identify bTB-positive cattle. Out of 512 cattle, only one animal was identified as a reactor, providing an estimated prevalence of 0.19% (95% Confidence Interval; 0–0.58%). The only positive reactor was found in Zakiganj upazila. As the prevalence of bTB in cattle in Sylhet appears to be low, it indicates that most of the upazilas of Sylhet district are free of the bTB infection. This prevalence is lower than the reported prevalence in other parts of Bangladesh. Thus, attempts should be made to maintain the current situation of bTB infection in cattle of Sylhet district.

## 1 Introduction

Bovine tuberculosis (bTB), an endemic disease in cattle in many countries, poses a global public health concern as its’ predominant pathogen, *Mycobacterium bovis*, causes zoonotic tuberculosis in humans that could be as fatal as typical tuberculosis, resulting in death if undiagnosed and untreated [[1], [2], [3], [4]]. The disease can be chronic and wasting in nature, which is characterized by progressive debilitation and the development of tubercles mainly in the lungs and infrequently in other organs depending on the portal of entry into the host body [5]. Due to the continuous production deficit with disease progression, trade prohibition of milk and milk products, and other costs for treatment and control programs, bTB provokes a huge economic impact in the affected countries and worldwide, with an estimated loss of USD 3 billion annually [5]. The transmission of the organism is fairly complex due to having a broad host range; usually, cattle, irrespective of age, get infected by direct contact with infected animals, or inhalation of aerosol produced by their coughing and sneezing, or even through ingestion of contaminated feed and water, while in calves, the disease is also transmitted by direct sucking of milk from infected cows [6,7]. Like other countries of the world, bTB is prevalent in different districts of Bangladesh with a substantial mortality rate [1,8,9]. The prevalence of bTB in cattle has been reported as 5.95%, 5.46%, 1.43%, and 7.78% in Dhaka, Chittagong, Mymensingh, and Sirajganj districts, respectively, as well as 33.73% in Rangpur division [[10], [11], [12]]. Studies also showed that the prevalence of the disease in the Red Chittagong breed and the breeding bull is 30% and 27.50%, respectively [13,14]. With the advancement of diagnostic methods at present, PCR is used to detect bTB in cattle besides several serological tests; nevertheless, the Caudal Fold Tuberculin (CFT) test is still recommended by OIE for screening purposes [3,15].

The study area Sylhet district, situated in the northeastern part of Bangladesh, has around 7 million cattle population comprising both indigenous and crossbreed mostly reared under a semi-intensive system [16]. Despite its known effect on cattle morbidity, mortality, and zoonotic significance, only a few epidemiological studies have been carried out focusing only on bTB in the Sylhet district of Bangladesh [1,10]. Most of these studies were in the vicinity of veterinary hospitals or covered only a small geographical area. Thus, information on the epidemiology of this disease in cattle remained limited in this region. Based on the pattern and clinical symptoms, expert opinions suggest that some of these morbidities and mortalities could be from bTB. However, like in other parts of Bangladesh, a systematic epidemiological study has been carried out to explore the epidemiological indices of this disease in this area. Thus, we designed the study to estimate the prevalence and spatial distribution of bTB in the Sylhet district.

## 2 Materials and methods

### 2.1 Study area

**Fig. 1:**
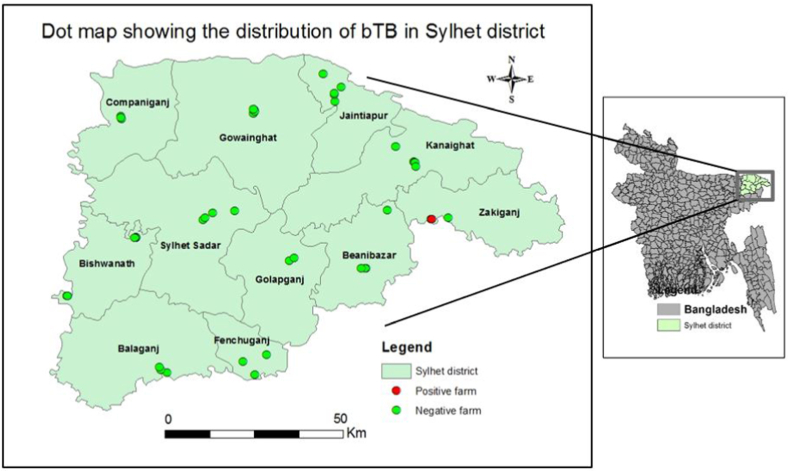
Dot map showing the positive case of bovine tuberculosis in Sylhet district identified in a cross-sectional study carried out from 1 September 2014 through 28 February 2015.

We conducted a cross-sectional study from 01 September 2014 through 28 February 2015 to determine the prevalence of bTB from selected farms of twelve upazilas (sub-districts) in the Sylhet district of Bangladesh (Fig. 1). Sylhet locates in the northeastern region of Bangladesh at 24.8917°N and 91.8833°E. The climate of Sylhet is humid subtropical with a predominantly hot and humid summer and a relatively cool winter. The district is within the monsoon climatic zone, with an annual average highest temperature of 23 °C (August–October) and an average lowest temperature of 7 °C (January).

### 2.2 Selection of the cattle farms

The target population of this study was cattle of any age belonging to the twelve upazilas of the Sylhet district. We collected a list of farms from the local Upazila Livestock Offices. Farms were selected from the list using a simple random sampling method. The inclusion criterion for selecting cattle farms was that the farms consisted of at least seven animals. Though we considered including five farms from each upazila, however, in some upazilas it was difficult to select five farms that fulfill the inclusion criterion. Only 48 farms from twelve upazilas fulfilled the requirement of inclusion criteria and hence were included in the study based on the consent of the owners for the participation, animal testing, as well as data collection (Fig. 1). These 48 farms had a total of 952 cattle (Supplementary File 1).

### 2.3 Sample size calculation and section of animals

The calculated sample size was 214 using the formula described by Humphry et al. [17] considering the expected prevalence as 5% [10], sensitivity and specificity of the CFT test as 50% and 99%, respectively [18], Z score at 95% confidence interval as 1.96, and desired absolute precision as 5%. To enhance reliability and ensure more statistical power, we randomly selected more animals than required that is 512 cattle among 952 from the selected farms through a simple random sampling technique.

### 2.4 Screening by Caudal Fold Tuberculin test (CFT)

All these selected cattle were tested with the CFT test using the standard procedure [12]. A hypodermic syringe was used to inject 0.1 ml of bPPD (bovine Purified Protein Derivative) intradermally under the skin of the caudal fold at the base of the tail. The immune system would react by producing inflammation at the injection site to cause swelling and/or skin discoloration if the animals are exposed to mycobacteria. The injection site was examined and palpated after 72 hours to examine whether there had been a reaction. The Field Disease Investigation Laboratory (FDIL), Sylhet provided bPPD for this study, which they obtained from the Istituto Zooprofilattico Sperimentale dell'Umbria e delle Marche, Perugia, Italy. We interpreted the results according to the OIE standard [15], that is, an animal was identified as a reactor and considered positive for bTB if it developed a visible or palpable swelling (≥4.0 mm) at the injection site.

### 2.5 Data collection and management

After diagnosing tuberculosis with the CFT test, we noted the numbers of positive and negative animals in the record book. The individual animal information was collected from the farmer's record books and also from a survey using a well-designed pre-tested questionnaire (Supplementary File 2). Owners of the selected farms were interviewed face to face for recording farm and cattle information using the same questionnaire. This questionnaire was especially focused on the information of individual animals as well as farm-level information to determine prevalence and determinants. We captured the geographical information as geographical coordinates by a global positioning (GPS) reader during sample collection. Collected cattle and farm information and test results were initially entered into a Microsoft Excel spreadsheet and coded for analysis (Supplementary File 3). We used Statistical Analysis System (SAS) version 9.4 to perform all statistical analyses. After initial descriptive analysis, some of the continuous variables like age of cattle and herd size were categorized due to their skewed distribution. The age was characterized as young (≤2 years) and older cattle (>2 years) arbitrarily, whereas the farm size was categorized as large (>25 cattle) and small farms (≤25 cattle) taking the mean of size as a cut-off. We created a few weighted variables by combining some information from the questionnaire. For example, the variable ‘hygienic condition’ was created by merging some variables related to farm hygiene practice. For each positive response to the question related to farm hygiene practice, the score was ‘1’ and for a negative response the score was ‘0’. The total score was calculated for each respondent by adding the score of each variable. The mean of the total scores of all respondents was calculated and used as a cut-off to create the new categorical variable ‘hygienic condition’, that is, if the combined score of a respondent was above the mean, then the farm was considered to have good hygiene. We also followed the same procedure was also to create the variable ‘management practice’. Information regarding all the variables under ‘hygienic condition’ and ‘management practice’ were available in Supplementary File 4.

### 2.6 Statistical analysis

We estimated the prevalence of positive results with a 95% confidence interval using the Proc SURVEYFREQ command in SAS. The difference in prevalence between individual animal traits and farm-level traits was tested with Fisher's exact test. We constructed prevalence and dot maps of bTB in ArcMap 10.1 and ArcCatalog 10.1 using geospatial data (latitude and longitude coordinates of selected farms) along with farm ID, upazilas, and test results collected from study sites.

## 3 Results

From 1 September 2014 through 28 February 2015, a total of 512 cattle were randomly selected from twelve upazilas in the Sylhet district. Among these 512 cattle, 36.32% were crossbred and 63.67% were indigenous cattle. In terms of gender, 351 (68.55%) were female and 163 (31.45%) were male (Table 1). The mean and standard deviation of the age of cattle tested were 3.24 and 1.53, respectively. The average number of cattle on farms was approximately 20 with maximum and minimum values of 91 and 07 cattle, respectively.

**Table 1:** Univariable analysis (Fisher's exact test) of plausible determinants of bovine tuberculosis obtained from a cross-sectional study in Sylhet district of Bangladesh.

Variables	No. of cattle tested (%)	No. of cattle positive (%)	*P*-value
Breed			0.70
Local	326 (63.67)	1 (100.00)	
Crossbred	186 (36.23)	0 (0.00)	
Gender			
Female	351 (68.55)	1 (100.00)	0.68
Male	161 (31.45)	0 (0.00)	
Age			
Older (>2 years)	373 (72.85)	1 (100.00)	0.61
Younger (≤2 years)	138 (27.15)	0 (0.00)	
Farm size			
Smaller (≤25 cattle)	363 (70.90)	1 (100.00)	0.64
Larger (>25 cattle)	149 (29.10)	0 (0.00)	
Calves kept together			
Yes	475 (92.77)	1 (100.00)	0.21
No	37 (7.23)	0 (0.00)	
Chronic coughing			
No	471 (91.99)	1 (100.00)	0.23
Yes	41 (8.01)	0 (0.00)	
Grazing facilities			
Yes	342 (66.80)	1 (100.00)	0.70
No	170 (33.20)	0 (0.00)	
Integrated farming			
No	146 (28.52)	1 (100.00)	0.20
Yes	366 (71.48)	0 (0.00)	
Presence of wildlife			
No	403 (78.71)	1 (100.00)	0.52
Yes	109 (21.29)	0 (0.00)	

Only one animal tested positive as a reactor among the 512 animals tested. Thus, the prevalence of bTB estimated in this study was 0.19% (95% confidence intervals; 0–0.58%). The single reactor was a local-breed female aged 4.5 years.

In univariable analysis, none of the determinants were statistically significant (p > 0.05) (Table 1). The dot map presented in Fig. 1 showed that the only positive animal belonged to Zakiganj upazila. No prevalence map was constructed due to the lack of positive cases in other upazilas.

## 4 Discussion

bTB is endemic in many countries [1] and is considered an emerging or re-emerging disease in some countries [19]. Adoptive veterinary control measures based on previous knowledge were capable of reducing the magnitude of this disease in several countries. The existence of bTB in Bangladesh has been reported in several studies [[10], [11], [12]]. However, information on virulence and epidemiological indices such as emergence, prevalence, spread, and persistence of infection in different hosts based on field observations were limited in the study area. Thus, the overall objective of this study was to investigate the epidemiology of bTB in cattle of the Sylhet district with a focus on the prevalence and geographical distribution. However, the result of the study showed that detectable reactors of bTB were very negligible, indicating no current or previous infection with *M. bovis* in cattle of the Sylhet district of Bangladesh. It implies that if bTB is present, despite the results of this study, the prevalence in cattle is at any rate low. In Sylhet, the farmed ruminant species cannot be considered epidemiologically separate populations as holdings having combined production with several species and sharing of pasture between various ruminant species are common practices. The presence of tuberculosis in beef cattle farms, goat farms, or sheep farms would presumably have led to infected dairy cattle as well, indicating that if the infection is present, the prevalence is probably low also in these populations.

Hossain et al. [10] conducted a study on the prevalence of bTB in several districts of Bangladesh including Sylhet where the estimate was low, 4.27%. Even this study reported zero prevalence of bTB in the Bogura district. The prevalence estimates in other districts were also low except in Tangail. Thus, the findings of this study support the findings of the current study.

In contrast, Rahman et al. [1] reported a higher prevalence (12.33%) of bTB in the Sylhet district than the estimate found in the present study. This variation in prevalence might be due to the differences in the sample (milk) and the test (PCR) used in this study. Another study showed a 33.73% prevalence of bTB by the CFT test in the Rangpur division of Bangladesh [12]. Norby [20] reported that the performance of bovine tuberculin skin tests is significantly affected by variations in agricultural zones and herd types. Thus, this discrepancy in prevalence could be due to the differences in the study population and agricultural regions.

It is a well-known fact for many infectious diseases including bTB that the risk of disease transmission increases with increasing population density [21]. Pabna and Sirajganj are known as the cattle-raising zones in Bangladesh and, thus, have a high cattle density. Mymensingh and Chittagong also have relatively high cattle density. On the other hand, Sylhet is known to have a low cattle density. Therefore, it is expected that the probability of animal-to-animal transmission of bTB was relatively less in Sylhet than in other parts of the country with high cattle density. Previous studies documented that the prevalence of bTB increased with increasing herd size [21]. The farm size of cattle in the study area is relatively smaller than in the other parts of the country. Hence, the probability of bTB infection might be lower in this area than in other parts of the country. It was also documented that local indigenous cattle are more resistant to bTB than exotic crossbreds [22]. A significant proportion of cattle included in this study were local indigenous (63%). In addition, most of the exotic crossbred cattle received genetic merits from the local indigenous ancestors, which might make them resistant to bTB. However, in a few studies, it was shown that the prevalence was higher in local cattle than in exotic crosses [12]. In a study, it was stated that recently infected cattle, cattle under stress due to malnutrition, gastrointestinal parasitic infestation, and other concurrent infections, and cattle with generalized TB would fail to react to the CFT test [23]. Stress due to starvation, long trekking, and other environmental stress, as well as ill health due to tick-borne diseases, heavy ectoparasitic infestation, and gastro-intestinal parasitic infestation were common in the present study. These could have been the reasons for the lower prevalence of bTB in this study.

The present study used the CFT test to screen out the positive reactor cattle in the study population. Picasso-Risso et al. [24] used a Bayesian approach and modeled the accuracy of the CFT test, where they found 80% sensitivity and 90% specificity. Due to this high sensitivity and specificity, the CFT test is recommended by OIE and is also widely used in the USA and New Zealand for screening purposes [25]. Moreover, Ragassa and Ameni [18] also calculated a high specificity, ranging from 94 to 99% in cattle. It implies that the CFT test is capable of identifying bTB-negative animals almost accurately. Thus, it can be stated that cattle identified as test negative in this study be declared bTB negative with little marginal error. However, the low sensitivity of this test indicates that this test is not well capable of identifying truly bTB-positive cattle, and this test may give a considerable number of false-negative reactions. As the estimated prevalence in this study was not adjusted for the sensitivity and specificity of the CFT test, the presented estimate was an apparent one. It indicates that the presented outcome in this study describes the almost true situation of bTB in cattle in the Sylhet district. The inclusion of young animals might incorporate a selection bias as testing younger animals had a lower prevalence of detectable bTB though Mondal et al. [26] did not find age as the significant risk factor in a study conducted in Mymensingh, Bangladesh.

## 5 Conclusions

This study indicates that the prevalence of bTB is low in the tested cohort. The local zebu cattle have better resistance or adopted tolerance to bTB, which may lead to less bTb infection resulting in low prevalence. Though the present study explores the very low prevalence of bTB in the local breed, further epidemiological investigation on disease burden, transmission dynamics, ecological and environmental factors, and the economic analysis of the effect of the disease could be undertaken by incorporating crossbred cattle to explore the complete epidemiology and ecology of bTB in Sylhet, Bangladesh.

## Funding statement

This research did not receive any specific grant from funding agencies in the public, commercial, or not-for-profit sectors.

## Data availability statement

Data is included in supplementary material in the article.

## CRediT authorship contribution statement

**Pradeep Kumar Mandal:** Data curation, Formal analysis, Investigation, Writing – original draft. **Md Irtija Ahsan:** Data curation, Formal analysis, Investigation, Writing – original draft, Writing – review & editing. **Hrithik Deb Apu:** Data curation, Formal analysis, Investigation. **Sharmin Akter:** Data curation, Formal analysis, Investigation. **Syed Sayeem Uddin Ahmed:** Conceptualization, Formal analysis, Supervision. **Suman Paul:** Conceptualization, Formal analysis, Methodology, Supervision.

## Declaration of competing interest

The authors declare that they have no known competing financial interests or personal relationships that could have appeared to influence the work reported in this paper.
