# Supplementary material for: Very low prevalence of bovine tuberculosis in cattle in Sylhet district of Bangladesh
Source: Heliyon. 2023 Nov 20;9(12):e22756. doi: 10.1016/j.heliyon.2023.e22756 (PMC10709486; doi:10.1016/j.heliyon.2023.e22756)
Supplement: Multimedia component 2 [file mmc2.docx]

**Unique Identification No.:**

**A Questionnaire for Prevalence and risk factors of Bovine Tuberculosis (bTB) in Sylhet District.**

**________________________________________________________________**

**Interview with cattle farmers**

**Conducted By:**


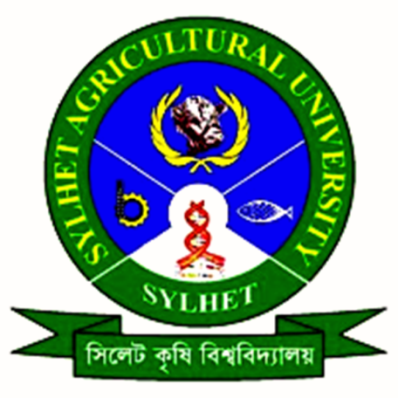


**Department of Epidemiology and Public Health**

In association with

**Department of Microbiology and Immunology**

**Sylhet Agricultural University,**

**Sylhet**

**Supported By:**

**Field Disease Investigation Laboratory**

**Sylhet**

**Objectives:**

- To determine the prevalance of Bovine Tuberculosis in Sylhet district.
- To identify the risk factors associated with Bovine Tuberculosis in Sylhet district.
- To study spatial distribution of Bovine Tuberculosis in Sylhet district.

**INFORMED CONSENT**

Addressing the farmer,

We are.....................................................................................................................

We are engaged in a research project by SAURes, Sylhet Agricultural University, Sylhet, and we have come from there. SAURes is attempting to study the prevalence and risk factors study of Tuberculosis (TB) in Cattle in all upazilas of Sylhet district.

Tuberculosis (bTB) is an infectious and zoonotically important disease of cattle that can markedly reduce the production and causes great loss of your farm. The result of this project will be of great benefit in the control and prevention of this fatal disease.

We select your farm and you are selected as an interviewee for this survey as a part of this project. We would like to ask you some questions related to our survey. It needs 30 minutes. You can skip any question if you are not interested to answer. Your given information will be kept confidential and your name will not be published in the report. We hope, you will join the interview because your information is very important for our survey.

Have you any more queries about us and survey? Yes No

Can we start interview now? Yes No

**A Questionnaire for Prevalence and risk factors of Bovine Tuberculosis (bTB) in Sylhet District.**

**Form: Cattle farmer**

**SURVEY COVER SHEET:**

| **IDENTIFICATION** |
| --- |
| **District:** ________________________________________________________  **Upazila:** ________________________________________________________  **Union:** _________________________________________________________  **Village:** _________________________________________________________  **Farm Name:**______________________________________________________ |

| **INTERVIEWER VISITS** | | |
| --- | --- | --- |
| **Name of the Interviewer** | **Date of 1^st^ visit** | **Date of 2^nd^ visit** |
| **1.**  **2.** |  |  |

| **Supervisor’s Name**  **____________________** | **Field Editor’s Name**  **__________________** | **Office Editor’s Name**  **___________________** |
| --- | --- | --- |

| **Beginning time :_________AM/PM** |
| --- |

**QUESTIONNAIRE FOR THE CATTLE FARMERS**

**Section-1: Background Information:**

1. **Name of the farm owner: :**____________________________________
2. **Age of the farm owner (years):**_______________
3. **Sex of the farm owner:** Male Female
4. **Educational status of the farm owner:**
   1. Illiterate b. Basic writing & reading c. Primary

d. JSC e. SSC f. HSC/ Diploma g. Degree and above

1. **Farm name:** ________________________________________
2. **Date of establishment of farm:**________________________________
3. **How did you (owner) start the farm business (multiple options possible)?**
   1. Bought the enterprise
   2. Bought cattle from other known dairy farms
   3. Bought animals from market without knowing their origin
   4. Gift
   5. Others specify: ________________________
4. **What is the purpose of your farm?**
   1. To produce products for home consumption only
   2. To produce products for market only
   3. To produce products for market and home consumption
5. **Is there any integrated farming in your farm?**

a. Yes b. No

1. **If yes, what type of integrated farming in your farm?**

a. Dairy & Beef farming b. Dairy & other domestic animals farming

1. **Herd size:_________**

**Section-2: Information related to possible risk factors for bovine Tuberculosis:**

1. **Information related to** **Management:**
2. **Housing system:**
3. Intensive (full time in shed)
4. Semi-intensive (some time of day in shed and other time in field)
5. Free range (full day in field and shed at night)
6. **Ventilation status of the barn/house:**

a. Poor b. Medium (satisfactory ventilation) c. Excellent

1. **Is there supply of balanced ration for the cattle of your farm?**

a. Yes b. No

1. **What is the source of water supply of your farm?**

a. Deep tube-well b. Pond c. River d. Other sources

1. **Are there any grazing facilities for your cattle?**

a. Yes b. No

**If yes what type of grazing system practiced?**

- 1. Rotational system b. strip system. c. set stocking system

**If yes, are cattle of your farm mixed with other domestic animals/wildlife during grazing?**

a. Yes b. No

1. **Is weaning of calves done in your farm?**

a. Yes b. No

1. **Are all calves kept together in your farm?**

a. Yes b. No

**If yes, what is the length of time calves kept together?**

a. Upto 6 months b. Upto 1 year c. Upto 1.5 year

**B. Information related to** **Bio security:**

1. **Is there is any footbath in your farm?**

a. Yes b. No

1. **Is Disinfectant regularly used in footbath?**

a. Yes b. No

1. **Is Disinfectant regularly used in farm premises?**

a. Yes b. No

1. **Is barn cleaned regularly?**

a. Yes b. No

1. **Is Disinfectant regularly used in barn?**

a. Yes b. No

1. **Is there proper drainage System for disposal of feces and urine?**

a. Yes b. No

1. **Are instruments properly cleaned and disinfected?**

a. Yes b. No

1. **Is there presence of wildlife around the farm?**

a. Yes b. No

1. **Sanitary condition of the calving site:**

a. Poor b. Medium (satisfactory condition) c. Excellent

**C. Information related to** **Disease:**

1. **Had any animal in your farm with chronic cough/chronic body wastage in the last six months?**

a. Yes b. No

1. **Is there any cattle suffered from TB in last two years?**

a. Yes b. No

1. **Have any cattle been tested with tuberculin/PPD before in your farm?**

a. Yes b. No

**If yes, how many cattle were tested as positive? ______**

**If yes, what happened with the cattle tested as positive (multiple options possible)?**

- 1. It remained at the farm
  2. It was slaughtered
  3. It was sold

1. **Is there any cattle died of TB in your farm?**

a. Yes b. No

**If yes, how did you dispose the carcass?**

- 1. Burial method b. Incineration c. Throw in open areas

1. **How do you get replacement stock (multiple options possible)?**
   1. My own farm by Artificial Insemination
   2. Insemination by own bull
   3. Purchasing from different cattle sources
   4. Others, specify: __________________

**D. Information related to** **Working staffs:**

1. **How many staffs are working in your farm?**
2. **Do staffs maintain personal hygiene?**

a. Yes b. No

1. **Any of the staffs working on your farm had tuberculosis?**

a. Yes b. No

1. **Any of the staffs working on your farm has tuberculosis at present?**

a. Yes b. No

**If yes, how many times does he visit the farm?______________**

**E. Individual information**

| **Sl. No.** | **Tag No.** | **Breed** | **Sex** | **Age** | **BCS** | **Tuberculin test Result** |
| --- | --- | --- | --- | --- | --- | --- |
|  |  |  |  |  |  |  |
|  |  |  |  |  |  |  |
|  |  |  |  |  |  |  |
|  |  |  |  |  |  |  |
|  |  |  |  |  |  |  |
|  |  |  |  |  |  |  |
|  |  |  |  |  |  |  |
|  |  |  |  |  |  |  |
|  |  |  |  |  |  |  |
|  |  |  |  |  |  |  |
|  |  |  |  |  |  |  |
|  |  |  |  |  |  |  |
|  |  |  |  |  |  |  |
|  |  |  |  |  |  |  |
|  |  |  |  |  |  |  |
|  |  |  |  |  |  |  |
|  |  |  |  |  |  |  |
|  |  |  |  |  |  |  |
|  |  |  |  |  |  |  |
|  |  |  |  |  |  |  |

**Section-3: Information related to the prevalence of bovine tuberculosis of the farm.**

**a. Numbers of cattle tested:______________________________________**

**b. Numbers of cattle tested as positive:_____________________________**

| **Finishing time :_________AM/PM** |
| --- |

**Thank You for your kind Information.**
