# Supplementary material for: Very low prevalence of bovine tuberculosis in cattle in Sylhet district of Bangladesh
Source: Heliyon. 2023 Nov 20;9(12):e22756. doi: 10.1016/j.heliyon.2023.e22756 (PMC10709486; doi:10.1016/j.heliyon.2023.e22756)
Supplement: Multimedia component 4 [file mmc4.docx]

**Information related to** **management practices:**

[Positive responses are marked with yellow color]

1. **Housing system:**
2. Intensive (full time in shed to avoid contact with other animals)
3. Semi-intensive (some time of day in shed and other time in field)
4. Free range (full day in field and shed at night)
5. **Ventilation status of the barn/house:**

a. Poor b. Medium (satisfactory ventilation) c. Excellent

1. **Is there supply of balanced ration for the cattle of your farm?**

a. Yes b. No

1. **What is the source of water supply of your farm?**

a. Deep tube-well b. Pond c. River d. Other sources

1. **Is weaning of calves done in your farm?**

a. Yes b. No

**Information related to farm hygiene practice:**

[Positive responses are marked with yellow color]

1. **Is there is any footbath in your farm?**

a. Yes b. No

1. **Is Disinfectant regularly used in footbath?**

a. Yes b. No

1. **Is Disinfectant regularly used in farm premises?**

a. Yes b. No

1. **Is barn cleaned regularly?**

a. Yes b. No

1. **Is Disinfectant regularly used in barn?**

a. Yes b. No

1. **Is there proper drainage System for disposal of feces and urine?**

a. Yes b. No

1. **Are instruments properly cleaned and disinfected?**

a. Yes b. No

1. **Sanitary condition of the calving site:**

a. Poor (not cleaned and disinfected)

b. Medium (irregularly cleaned and disinfected)

c. Excellent (regularly cleaned and disinfected)
